# The Role of Prefrontal Cortical Surface Area and Volume in Preclinical Suicidal Ideation in a Non-Clinical Sample

**DOI:** 10.3389/fpsyt.2019.00445

**Published:** 2019-06-21

**Authors:** Sahil Bajaj, Adam C. Raikes, Ryan Smith, John R. Vanuk, William D. S. Killgore

**Affiliations:** ^1^Social, Cognitive and Affective Neuroscience Laboratory (SCAN Lab), Department of Psychiatry, College of Medicine, University of Arizona, Tucson, AZ, United States; ^2^The Laureate Institute for Brain Research (LIBR), Tulsa, OK, United States

**Keywords:** cortical structure, stress, social support, neuroanatomy, personality assessment

## Abstract

Suicidal ideation (SUI) can occur in the absence of concomitant psychiatric diagnoses, and even normal levels can be problematic among individuals experiencing excess stress or lack of social support. The objective of this study was to investigate the neuroanatomical basis of SUI in non-clinical human populations who are within the normal limits of SUI, after accounting for elevated stress and perceived lack of social support. Neuroanatomical data were collected from 55 healthy individuals (mean age 30.9 ± 8.1 years, 27 females) whose depression severity levels were below the *Diagnostic and Statistical Manual of Mental Disorders* criteria. Measures of SUI, aggression, stress, non-support, and treatment rejection were collected from the treatment-consideration scales (TCS) of the Personality Assessment Inventory (PAI). Correlations between standardized SUI scores and three brain morphometry measures, *including* vertex wise cortical thickness (CT), cortical surface area (CSA), and cortical volume (CV), were estimated for each participant, controlling for age, sex, intracranial volume, and the remaining TCS measures. We observed a significant negative association between scores on SUI and both CSA and CV (cluster-forming threshold of *p* < 0.005, clusterwise threshold of *p* < 0.05, *FDR* corrected for multiple comparisons) within the left rostral middle frontal gyrus. Our findings suggest that greater CSA and CV within the dorsolateral prefrontal cortex are associated with reduced SUI in a non-clinical population with mild levels of stress and perceived lack of social support. Because the dorsolateral prefrontal cortex has been broadly linked to cognitive reappraisal, self-critical thoughts, and emotional regulation, greater CSA and CV within these regions may lead to better mental health by protecting healthy individuals from engaging in SUI during periods of stress and perceived insufficient social support. As our data consisted of only healthy individuals with non-clinical levels of SUI, further investigation will be necessary to explore the neural basis of SUI in populations who may be at greater risk of future suicidal behavior

## Introduction

Approximately 150,000 people in Europe die because of suicide every year (1), and the percentage of annual suicide attempts in the United States has reportedly increased significantly from 0.62% to 0.79% (from the sample recruited between 2004 and 2005 and between 2012 and 2013) among individuals aged 21 years and older (2), making suicide one of the primary causes of death (3). On the whole, approximately 45,000 Americans and 800,000 people worldwide commit suicide each year, the 10th and the 17th leading cause of death, respectively (https://afsp.org/about-suicide/suicide-statistics/, http://www.who.int/en/). Furthermore, 80% of individuals who commit suicide show no symptoms during their most recent contact with a healthcare professional (4). However, despite the high prevalence of risk factors for suicide (e.g., depressive disorders, bipolar disorders, anxiety disorders, personality disorders, and factors such as aggression, impulsivity, hopelessness, and heredity) within the general population, only a minority of individuals from the general population commit suicide. Because of the relatively small proportion of successful suicides in such population, it is exceptionally difficult to prospectively identify specific individuals who are likely to (successfully) attempt suicide (5).

There is increasing interest among clinical researchers to better understand the neuroanatomical factors associated with suicidal behavior (6, 7); however, due to the early state of knowledge in this area, and a variety of complicated interactions with other variables (e.g., psychiatric disorders, medical and family history, substance abuse, and social and emotional factors) (8), there is currently a lack of consensus within neuroimaging studies investigating the neuroanatomical basis of suicidal ideation (SUI) or completed suicide. In recent years, a number of cortical measures, such as cortical thickness (CT), cortical surface area (CSA), and cortical volume (CV) have been used to assess different facets of brain morphology, which are known to relate to specific brain function (9, 10). For instance, neuroanatomical differences, i.e., reduced volume within the frontal lobe (11, 12), have been associated with suicide or suicidal behavior, including suicide attempts. Frontal lobe lesions have also been linked to impulsive mood and poor decision-making (13, 14). The orbitofrontal cortex (OFC) and amygdala are key regions linked to emotion and impulse regulation, with work suggesting that structural abnormalities within these regions can potentially influence these functions and increase the risk for suicidal behavior (15). Thoughts of death are also associated with reduced CT in frontoparietal regions and insula, as well as widespread differences in white-matter fractional anisotropy and radial diffusivity (16). Moreover, prefrontal regions play an important role in cognitive reappraisal processes, which are important for regulating dysfunctional emotional states. Prior anatomical findings also suggest that there are direct connections between lateral portions of prefrontal cortex (lPFC) and the amygdala and that the lPFC contributes to the modulation of the amygdala during cognitive reappraisal (17). From a clinical point of view, Baeken and colleagues recently explored the SUI attenuation following 4 days of a high-frequency brain stimulation procedure, called accelerated intermittent theta burst stimulation (aiTBS) (18). In that study, high perfusion patterns within the default-mode network were associated with high baseline levels of SUI, and aiTBS treatment reduced perfusion within the bilateral frontopolar cortices and decreased SUI. Magnetic seizure therapy has also been shown to completely resolve SUI in 44% of individuals by inducing frontal cortex neuroplasticity (19).

Given the sobering statistics related to suicidal behavior and the current lack of identified external prodromal cues and lack of new methods for early detection of at-risk individuals, there is a critical need to better understand the neurobiological basis of cognitive patterns that might point toward worsening suicidal tendencies. One approach that has been neglected thus far is to identify brain behavior patterns that are protective against or point toward potential suicidal tendencies in otherwise healthy/non-clinical individuals. In particular, a better understanding of the neurobiological associations with pre-clinical/normal to minimal suicidal thoughts in the general healthy population may provide important insights into potential risk factors for early, pre-clinical, SUI, or future suicide attempts as well as any protective role that specific patterns of brain organization may confer during periods of perceived stress or lack of social support. This is because the majority of the prior research has focused on the individuals who have experienced actual suicidal intent or engaged in suicidal behavior, rather than focusing on the population experiencing suicidal thoughts without ever intending to carry out such an act (i.e., passive thoughts such as “I wish I would not wake up tomorrow”). Consequently, very little is known about the brain organization and structural morphometry associated with suicidal thinking among populations without overt psychopathology.

The primary focus of the present study was to use the cortical measures to extend previous work by studying non-clinical individuals from the general population (who nevertheless may be experiencing mild stress and insufficient social support) and correlate these measures with mild pre-clinical indicators of SUI. For that, we estimated whole brain vertex-wise CT, CSA, and CV in these individuals and explored their relationship with SUI on a standardized assessment measure. We hypothesized that after accounting for factors that are known to contribute to suicidal potential index (20) *such as* aggression, stress, and perceived lack of social support, greater CT, CSA, and/or CV for regions within the prefrontal cortex would be associated with better mental health, as evidenced by lower levels of SUI.

## Materials and Methods

### Participants

Neuroanatomical data were collected from 55 participants, who were between 18 and 45 years of age (mean age = 30.9 ± 8.1 years, 27 females; 28 males). All participants were recruited between 2010 and 2013 from the greater Boston region *via* posted flyers and Internet advertisements seeking healthy normal individuals to participate in a study of emotional intelligence and brain functioning. All interested volunteers were screened *via* a brief telephone interview that included questions about medical and general psychiatric history, substance use, and contraindications for magnetic resonance imaging. However, no specific clinical screening was conducted for SUI or attempts prior to entry. Participants were screened for any evidence of past or present psychotic, depressive, or medical disorders using a structured series of questions adapted from the Structured Clinical Interview for *Diagnostic and Statistical Manual of Mental Disorders* (DSM-IV), text revision (SCID-I) (21). Participants reporting evidence of a history of DSM-IV Axis I mental disorders (major depression, eating disorder, psychotic experiences, social anxiety, or obsessions/compulsions), excessive substance use, drug or alcohol treatment, or severe medical or neurological conditions, or having contraindications for scanning (e.g., metal in the body or pregnancy) were excluded from the study. A total of 173 participants completed initial phone screens, 70 were eligible and enrolled in the study, and 6 were withdrawn prior to completion for various reasons (e.g., deliberate falsification of identity, poor eyesight, or suspect motivation). Another 9 had incomplete personality assessment scores or neuroimaging data due to technical issues, leaving a total of 55 usable datasets. It should be noted that the primary goal of the funded grant which sponsored this study was to identify neuroimaging correlates of emotional intelligence in healthy individuals and to address the need to develop psychological resilience among Service members and their families to promote well-being and prevent behavioral health outcomes. Therefore, SUI data reported in the present study were collected from healthy individuals. Some behavioral data from this sample have been reported elsewhere (22–25), but the associations between SUI and brain morphometry are novel and have never been published. Any data not published within this article will be made available by reasonable request to the senior author (WDSK). All participants provided written informed consent prior to enrollment. The study protocol was approved by the Institutional Review Boards of McLean Hospital and Partners Healthcare (2009-P-002230), and the U.S. Army Human Research Protections Office (Log Number: A-15731).

### Data Acquisition

*Brain anatomical data*. We recorded T1-weighted magnetic resonance imaging (MRI) data using a 3-Tesla Siemens TIM Trio whole-brain MR scanner located at the McLean Hospital Imaging Center. Before the scan, each participant was instructed to rest, relax, and try his/her best to minimize movement during the entire scan. Head movement was minimized with foam padding placed comfortably about the head. T1-weighted data for each participant were acquired using a 3D magnetization-prepared rapid acquisition gradient echo (MPRAGE) sequence, which consisted of 128 sagittal slices [slice thickness = 1.33 mm, voxel resolution = 1.33 × 1 × 1 mm, field of view (FOV) = 256 mm with repetition time/echo time/flip angle/inversion time of 2,100 ms/2.25 ms/12°/1,100 ms].

*Personality assessment inventory (PAI).* The PAI is a self-report scale used to detect and quantify adult psychopathology, including anxiety, depression and mania, interpersonal styles, and treatment-related issues, which are important in the diagnosis of various psychiatric disorders (26, 27). The PAI has been shown to have good validity and reliability with a high degree of internal consistency (median alpha and test–retest correlations exceed 0.80 for the 22 scales) (28). A trained research technician, supervised by a licensed neuropsychologist, administered the PAI to each individual. The PAI includes 22 non-overlapping scales, which are aggregated into four factors (validity, clinical, treatment consideration, and interpersonal scales) (29). Given the current study’s focus on suicidal behavior, we restricted our analyses to the SUI subscale of the treatment-consideration scale (TCS). Other sub-scales of the TCS such as aggression (AGG), stress (STR), and non-support (NON), which could be associated with suicide potential (30), were used as covariates.

Participants were asked to rate both the frequency and severity of potential indicators of SUI ranging from hopelessness to general and vague or concrete plans for a suicidal act. Raw data were converted to standardized T-scores with a mean of 50T and a standard deviation of 10T (26, 29). PAI scores greater than 50T indicate that the participant endorsed the relevant items to a greater extent than typical for their normative age group, with higher scores indicating greater deviation from normal (31). Scores below 60T are considered to be within normal limits for SUI, while scores between 60T and 70T are considered moderate SUI (32). Scores above 70T are interpreted as significant SUI and are rarely encountered in the general population. For the covariates, scores below 60T reflect reasonable control over aggression (AGG); stable, manageable stress levels (STR); and reasonable social support (NON). However, scores between 60T and 70T reflect individuals who may be impatient, irritable, and quick-tempered (AGG), experiencing a moderate degree of stress due to difficulties in some major life area (STR), or experiencing a moderate degree of perceived non-support from friends, loved ones, and society (NON). Scores above 70T are interpreted as consistent with significant aggression, stress, and perceived non-support from society.

### Data Analysis

*Preprocessing.* Raw neuroanatomical data were visually inspected for each participant. We used the standard “recon-all” pipeline in FreeSurfer 6.0.0 (https://surfer.nmr.mgh.harvard.edu) to process the neuroanatomical data for all the participants. The basic preprocessing pipeline included intensity normalization, removal of non-brain tissue, automated transformation to the standard MNI co-ordinate system, volumetric segmentation into cortical and sub-cortical matter, and cortical segmentation of the cerebral cortex (33). In order to improve the signal-to-noise ratio (SNR) and detect larger effects, brain images were smoothed using 12 mm full-width at half maximum (FWHM) Gaussian kernel. FreeSurfer’s preprocessing accuracy was inspected using standard quality control steps, which involved a careful visual inspection of skull-stripped brain volumes, masks, and pial surfaces. None of the participants were excluded after performing the above mentioned standard quality control steps. The PAI-SUI data were also screened for outliers using SPSS 22 (https://www.ibm.com/analytics/us/en/technology/spss/). For this manuscript, the cutoff value chosen for PAI-SUI was 59T. Four participants who scored greater than 59T on the SUI-scale were also identified as outliers (i.e., with a value more than 1.5 inter-quartile range above the upper quartile) and were excluded from data analysis. None of the remaining 51 participants were identified as outliers on the AGG, STR, or NON scales.

*Association between brain morphometry and SUI scores.* Three structural measures (CT, CSA, and CV) were estimated separately for the left and the right hemispheres for each participant. CT and CSA were estimated using conventional methods (34, 35). However, the conventional method for estimating CV involves the multiplication of CSA by CT at each vertex (36), which may lead to either over- or under-estimation of the cortical measures of that specific tissue (37). Therefore, we estimated CV by defining an oblique truncated triangular pyramid using three vertices in the white surface and three matching vertices in the pial surface (37). Details about this recent methodology can be found in a recently published manuscript by Winkler et al. (37). Maps of CT, CSA, and CV of the brain of each participant were created using the FreeSurfer processing pipeline. We fit individual general linear models (GLMs) to the left and right hemispheres using FreeSurfer’s statistical engine (mri_glmfit) to estimate the relationships between SUI and raw CT, CSA, and CV. Each of the cortical measures [i.e., raw CT, CSA, and CV (dependent variables)] was regressed on SUI (independent variable), controlling for age, sex, AGG, STR, and NON. Intracranial volume (ICV) was used as an additional covariate for correcting CSA and CV. To determine robust effects in morphometric analyses, a minimum cluster-wise threshold (CWT) of *p* < 0.05 at cluster-forming threshold (CFT) of *p* < 0.005 with FWHM > 10 mm was recommended (38). Therefore, in the present study, we used a CWT of *p* < 0.05 and CFT of *p* < 0.005 at FWHM = 12 mm (FDR-corrected for multiple comparisons using Monte Carlo simulations and corrected for both hemispheres). In addition, we used an even more stringent CFT of *p* < 0.001 for reporting significant clusters that showed associations with SUI (two-tailed).

## Results

### Descriptive Statistics

**Figure 1:**
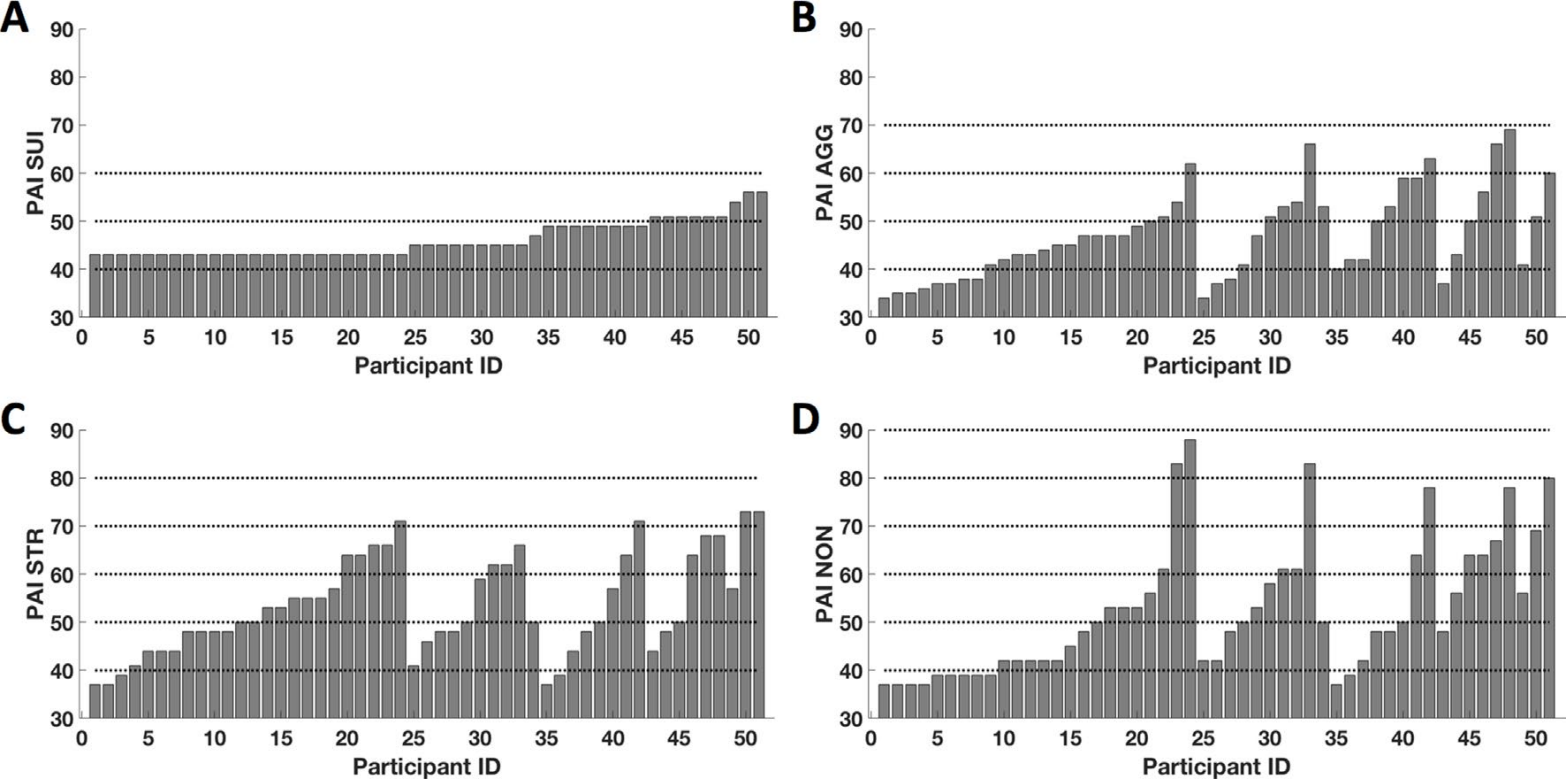
Subjectwise distribution of T-scores from the Personality Assessment Inventory–treatment-consideration scales (PAI-TCS). Here we show subjectwise distribution of T-scores for PAI-suicidal ideation (SUI) **(A)**, PAI- aggression (AGG) **(B)**, PAI- stress (STR) **(C)**, and PAI-non-support (NON) **(D)**. Scores are sorted from minimum to maximum for PAI-SUI and corresponding PAI-AGG, PAI-STR, and PAI-NON. Here, dotted lines represent the reference lines at T-scores of 40T, 50T, 60T, 70T, 80T, and 90T.

After excluding four outliers, raw SUI scores ranged between 43T and 59T (mean = 46.04 ± 3.8) (**Figure 1A**). In other words, this was generally a non-clinical sample. On average, participant responses on the AGG scale (mean = 47.10 ± 9.2) ranged from minimal to significant evidence of aggression, with 11 participants (21%) scoring between 50T and 60T, and 6 participants (12%) scoring more than 59T (**Figure 1B**). On the STR scale, participant responses (mean = 53.41 ± 10.3) ranged from minimal to significant levels of stress, with 9 participants (18%) scoring between 50T and 59T, and 15 participants (29%) scoring more than 59T (**Figure 1C**). Lastly, participant perceptions of insufficient social support (mean = 52.53 ± 13.9) ranged from minimal to significant, with 8 participants (16%) scoring between 50T and 59T and 14 participants (27%) scoring more than 59T (**Figure 1D**). Although the mean T-score for AGG was within the normal limits, the mean T-scores for STR and NON were at the mild level (i.e., with T-scores above the mean compared to scores of individuals in the standardized sample); however, in total, 23 participants (45%) showed significant endorsement of items consistent with aggression, stress, or non-support. The subject-wise distribution of T-scores from all PAI-TCS is shown in **Figure 1** (sorted from low to higher levels of SUI, followed by AGG, STR, and NON).

### Association Between SUI and Cortical Structure

**Figure 2:**
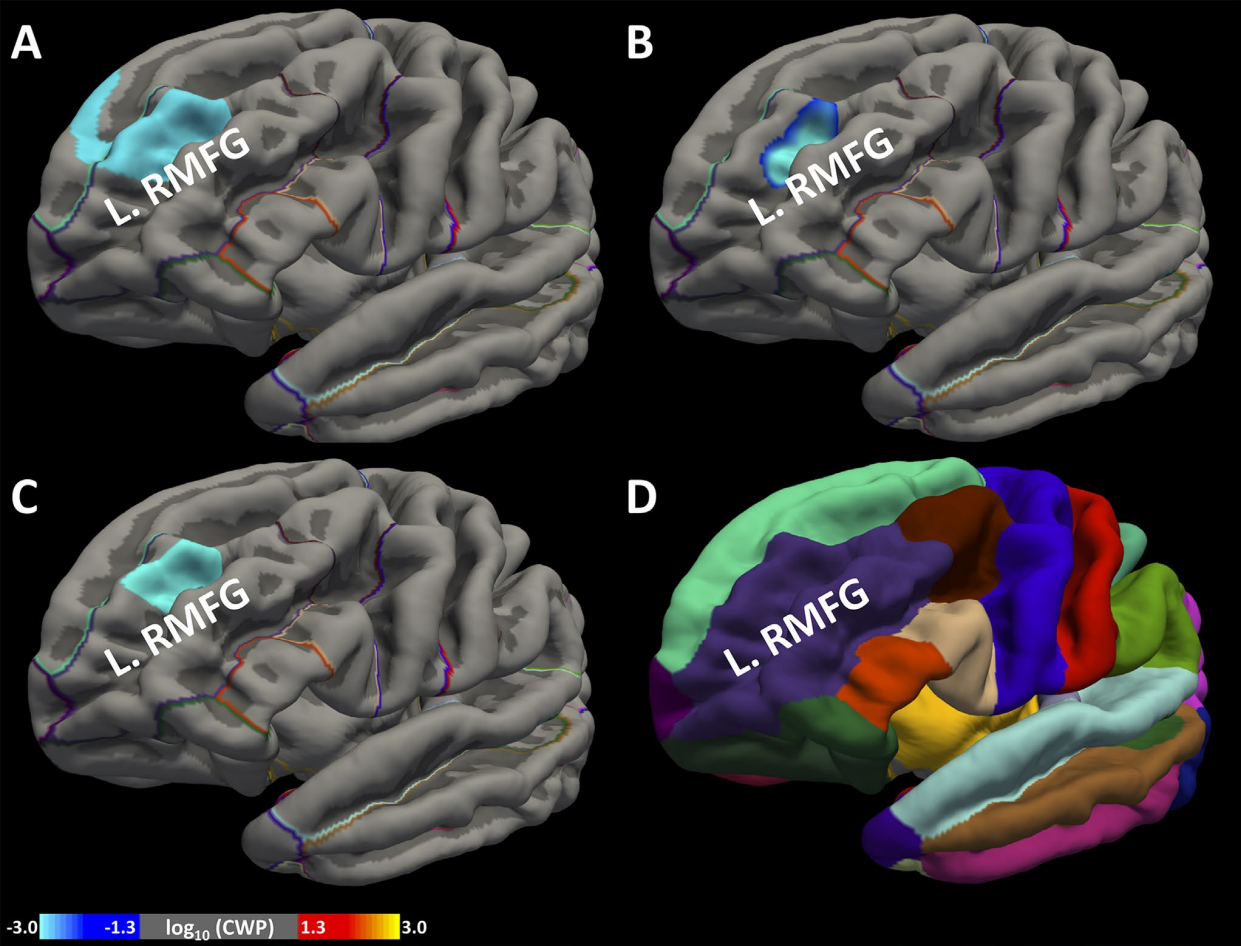
Association between greater cortical surface area (CSA), cortical volume (CV), and SUI. We identified a region within the frontal lobe the left rostral middle frontal gyrus (L.RMFG), which showed a significant association of greater CSA **(A)** and greater CV **(B)** with lower SUI at cluster-forming threshold (CFT) < 0.005. We also found the same cluster showing significant association of greater CSA **(C)** with lower SUI at CFT < 0.001. Standard anatomical location of L.RMFG is shown in Desikan atlas **(D)**. Colorbar represents the distribution of logarithm of *p* values.

*At a CWT of p* < 0.05 *and CFT of p* < 0.005: We found a cluster with its peak in the left rostral middle frontal gyrus (L.RMFG), which is part of the dorsolateral prefrontal cortex, showing a significant negative association between SUI and CSA (**Figure 2A**), as well as between SUI and CV (**Figure 2B**). This cluster also spanned across some portions of the superior frontal gyrus. However, we did not find a significant association between SUI and CT.

*At a CWT of p* < *0.05 and CFT of p* < *0.001*: At the more stringent CFT, again we found a cluster with its peak in the L.RMFG showing a significant negative association between CSA and SUI (**Figure 2C**). However, we did not find a significant association between SUI and CT or CV.

The preceding findings are summarized in **Table 1**, and the location of L.RMFG in the Desikan atlas (33) is shown in **Figure 2D**.

**Table 1:** Brain clusters showing a significant association between cortical surface area (CSA), cortical volume (CV), and suicidal ideation (SUI).

Clusters showing significant relationships between SUI, CSA, and CV						
Cluster number	Maxima	Peak co-ordinates (MNI: *X*, *Y*, *Z*)	CWP	Number of vertices within the cluster	Cluster size (mm^2^)	FreeSurfer label
*At CWP < 0.05, CFT of p < 0.005* (*FDR* corrected for multiple comparisons using Monte Carlo simulation)						
1	−3.65	−22.6, 49.5, 21.3	0.001	1544	1025.46	L. RMFG (for SUI vs. CSA)
2	−3.42	−24.8, 49.1, 15.6	0.020	681	449.50	L. RMFG (for SUI vs. CV)
*At CWP < 0.05, CFT of < 0.001* (*FDR* corrected for multiple comparisons using Monte Carlo simulation)						
1	−3.65	−22.6, 49.5, 21.3	0.007	567	408.16	L. RMFG (for SUI vs. CSA)

**Figure 3:**
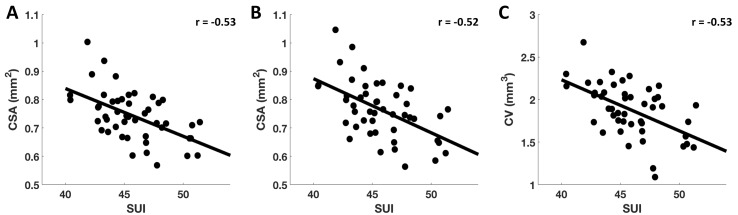
Correlation between SUI and estimated CSA and CV. After extracting subjectwise measures of SUI, CSA, and CV, here, we demonstrate significant negative correlations found between SUI and CSA at CFT of *p* < 0.005 **(A)** and CFT of *p* < 0.001 **(B)**, and between SUI and CV measures at CFT of *p* < 0.005 **(C)** for L.RMFG.

Also, for visualization purposes, we extracted CSA and CV measures within the above reported clusters for each participant and plotted them against SUI. Data points with Cook’s distance of more than 3 times the mean were considered as outliers and were excluded from the scatter plots. The observed negative partial correlations [with age, sex, ICV, and TCS (i.e., AGG, STR, and NON) as covariates] between SUI and CSA at CFT of *p* < 0.005 (*r* = −0.53) (**Figure 3A**) and at CFT of *p* < 0.001 (*r* = −0.52) (**Figure 3B**), and between SUI and CV at CFT of *p* < 0.005 (*r* = −0.53) (**Figure 3C**) within the L.RMFG, are shown in **Figure 3**.

## Discussion

In this study, we explored the association between pre-clinical SUI and measures of cortical structure within a non-clinical sample of individuals who did not meet DSM-IV criteria for Major Depressive Disorder (MDD) but had mild-to-moderate symptoms of stress and perceived non-support. We identified a cluster with a peak in the left rostral middle frontal gyrus that indicated greater CSA and CV in those with lower SUI scores. This suggests that greater surface area and volume within this region of the dorsolateral prefrontal cortex may be associated with better mental health, as evidenced by reduced preoccupations or thoughts of death and suicide within a non-clinical sample. These findings show a potentially protective role of frontal brain areas against cognitions that are often associated with increased suicidal thinking. Since the frontal brain areas have been reported to be strongly associated with cognitive reappraisal, self-critical thoughts, and emotional regulation, we, therefore, suggest that similar or comparable areas may play a crucial role to predict severe levels of SUI in clinical populations. Our study is novel, as it focused on identifying an association between measures of cortical structure and suicidal thoughts among individuals without overt evidence of past or current psychopathology, a topic that has received little attention but that may have relevance for understanding the pre-clinical or prodromal stages of suicidal thinking. Identifying potential risk factors that may predispose an individual to progress toward more severe psychopathology in the future is necessary in order to develop effective preventative interventions that aim to reduce the symptom presentation and evolution. Moreover, these findings emphasize the important role of the dorsolateral prefrontal cortex in maintaining healthy cognitive and emotional perspectives and potentially regulating thought, affect, and behavior.

We showed that at stringent cluster-forming and cluster-wise thresholds, there was a significant negative association between L.RMFG morphometry and SUI scores. Previous whole-brain analyses have shown that, compared to healthy controls, young individuals (attempters and non-attempters) with current SUI, assessed with the Columbia-Suicide Severity, had significantly less CV within the L.RMFG (39), a region corresponding to that found here. Our findings extend this prior work by illustrating a similar relationship in a non-clinical group within the normal range of SUI, after accounting for perceived stress and lack of support. Here, greater volume and CSA of the L.RMFG was associated with fewer suicide-related thoughts. Our findings are also consistent with a number of functional activation studies of SUI and behavior. For instance, Thompson and colleagues showed an association between activation within left frontal regions and suicidal behavior and found that the individuals with a high risk of suicidal thoughts and actions had reduced brain activity in the left frontal regions during the emotional Stroop task (40). Gosnell and colleagues also suggested that reduced volume within the frontal lobe (and within the temporal lobe) may be an important risk factor for suicidal thoughts or behavior (12). It should be noted that it was CSA and CV, but not CT, which showed significant association with pre-clinical SUI within a non-clinical sample of individuals. Mathematically, CV is the product of CT and CSA; therefore, both of these measures, i.e., CT and CSA, contribute to the measures of CV (36, 37). In other words, the sensitivity of CV accounts for CT as well as the CSA. However, compared to CT, CSA contributes more to the measures of CV (41). Therefore, it was not surprising to find an association between minimal levels of SUI and both CSA and CV, but not between minimal levels of SUI and CT. Together, these findings suggest that the dorsolateral regions of the prefrontal cortex may play a critical role in regulating emotion and cognitions related to potentially self-destructive outcomes.

There are three potential interpretations of our findings that will require further exploration. First, our findings may indicate that larger surface area and CVs within the observed areas of the prefrontal cortex are protective against negative thoughts in healthy individuals. This interpretation would suggest that greater surface area and volume within this region may contribute to 1) a greater capacity to regulate the emotional responses that lead to SUI; 2) greater ability in re-appraising situations in a healthier manner, or 3) potentially in inhibiting thoughts about self-harm or inhibiting impulses to engage in such behaviors. Second, the present findings are based on self-report, so it is also possible that the observed levels of SUI considered typical in a non-clinical population, in the presence of elevated stress and non-support scores, may reflect a possible denial of existing suicidal proclivities. Regardless, greater CSA and CV appear to be associated with reduced SUI, even when accounting for levels of stress and perceived lack of social support. A third, but perhaps less tenable, interpretation is that the normal thoughts related to suicide (and promoting factors) could instead lead to reduced CSA and CV even at low levels in healthy individuals. Of course, it is also possible that the observed association between SUI and cortical structure is non-causal in either direction and reflects the influence of some third unknown factor. However, a causal link between middle frontal structure and SUI appears plausible in light of the well-established role of the middle lateral prefrontal regions in action planning, behavioral control, and cognitive reappraisal processes (42–44), and previous findings that reduced volume of this region has been associated with SUI in clinical samples (39).

In addition, because the rostral middle frontal cortex is known for its involvement in passive maintenance and uninstructed generation of negative emotions (45, 46), our findings could suggest a link between decreased area and volume in this region and a predisposition toward maladaptive emotion-driven behavior. Disrupted function within prefrontal networks could perhaps impair decision-making and play a role in modulating the cognitive processes associated with carrying out suicidal acts (47, 48). Clinically, such suicidal cognitions have been associated with hopelessness about the future, difficulty in controlling emotional responses, and a tendency to choose suicidal acts over other alternatives (47). Functional MRI studies have also shown the involvement of middle frontal brain regions during reappraisal and self-criticism (49). Given these considerations, greater area and volume in this region could perhaps be protective against mental health concerns that involve consideration of self-destruction as a solution to immediate pains or struggles. We believe that our findings may contribute to further understanding of potentially similar morphometric behavior associations in more clinically severe cases.

### Strengths and Limitations

This study benefits from using a whole-brain surface-based morphometry approach to estimate vertex-wise cortical estimations. In particular, it should be noted that even a larger smoothing kernel size in surface-based analysis, unlike volume-based analysis, never extends into bone/air/white matter. In addition, whole-brain vertex-wise cortical estimations do not bias findings toward a specific set of brain areas, as compared to region- or specific hypothesis-based approaches (25, 50). Second, we used stringent cluster-forming thresholds of *p* < 0.005 and *p* < 0.001 to determine significant effects. Therefore, our analysis methods likely minimized the possibility of false-positive findings. Despite the aforementioned strengths, the findings of this study should be interpreted in light of several limitations. As our study involved neuroanatomical data of moderate resolution from a relatively small to moderate size sample and was focused on only three cortical measures, future studies would benefit from the use of high-resolution (<1 mm isotropic voxel size) structural imaging data from a larger sample size and should include other cortical measures (e.g., cortical folding) to further investigate the association between brain structure and SUI. Also, while many of the suicide items from the PAI were focused on the present occurrence of suicidal thoughts, some items can be interpreted in an open-ended way regarding the indexed time frame. It is therefore unclear to what degree our findings are influenced by the temporal recency of suicidal thoughts. Future research using more fine-grained measures of suicidal cognitions will be necessary to obtain a more detailed picture of the association between brain structure and SUI in non-clinical populations. Finally, the present study was only focused on non-clinical individuals with mild to moderate symptoms of stress and perceived non-support, and it is therefore not possible to generalize these findings to clinically significant cases of SUI. Consequently, future work would benefit from extending these methods to clinical samples in order to determine whether the observed trajectory continues at moderate to severe levels of SUI.

## Conclusions

The present findings showed that greater CSA and CV within a specific brain region in the middle frontal cortex, which has previously been linked to cognitive reframing, reappraisal, and action planning, may play a role in protecting healthy individuals from SUI. Our findings suggest that estimations of morphometric measures may help to better understand the brain basis of suicidal thoughts and behaviors more generally, and that differences in cortical structure in this specific region could perhaps serve as a potential risk/protective factor related to potential suicidal cognitions (as well as a potential target for treatment and prevention). This region appears to play a critical role in some aspects of mental health, and larger volume of this region appears to be associated with a reduced tendency to focus on thoughts associated with self-harm. Future research would benefit from using longitudinal study designs to investigate whether cortical area or volume could aid in predicting the likelihood of future SUI or suicide attempts, or whether recurrent SUI instead brings about alterations in cortical structure.

## Ethics Statement

The study protocol was approved by the Institutional Review Boards of McLean Hospital and Partners Healthcare, and the U.S. Army Human Research Protections Office.

## Author Contributions

SB analyzed the data and wrote the initial draft. AR and RS helped with data analysis and contributed to the writing of the initial draft. JV contributed to the writing of the initial draft. WK designed and supervised all aspects of the study and contributed to writing of the manuscript.

## Funding

This research was supported by a grant from the U.S. Army Medical Research and Materiel Command to WDSK (W81XWH-09-1-0730). The opinions, interpretations, conclusions, and recommendations in this paper are solely those of the authors and are not necessarily endorsed by the Department of Defense or the U.S. Army Medical Research and Materiel Command.

## Conflict of Interest Statement

The authors declare that the research was conducted in the absence of any commercial or financial relationships that could be construed as a potential conflict of interest.
